# Factors Influencing Location of Death

**DOI:** 10.1093/geroni/igaf122.2316

**Published:** 2025-12-31

**Authors:** Lauren Montemuro Rode

**Affiliations:** Bryn Mawr College, Bryn Mawr, Pennsylvania, United States

## Abstract

This research aims to explore contributing factors to location of death, with a focus on how the social network can impact where end-of-life (EOL) occurs. This analysis includes four primary locations of death: home, hospital, nursing home/assisted living facility (NH/ALF), and hospice in-patient center (IPC). The research theoretically draws from Life Course Perspective, which names the various influences of biological, psychological, and sociological spheres, as well as The Convoy Model, which specifically names consideration of closeness, quality, function, and structure when analyzing a network. The analysis includes demographic variables, health related variables, and social network variables through progressive modeling. A multinomial logistic regression analysis is applied, using longitudinal data from the Health Retirement Study ranging from 2000 through 2018 (maximum sample size of 10,414). Social Network Factors demonstrate the largest impact related to likelihood of EOL in SC/ALF vs home. Larger household size, increased number of children, discussing EOL wishes, increased time spent with children, and higher frequency of religious service attendance all decreased likelihood of EOL in SC/ALF relative to home. Length of time of terminal illness and heart, circulatory, and blood condition as COD (reference group, Cancer) appears to be a common barrier to death at home (vs hospital, SC/ALF, and hospice IPC). Household size is a consistent support to death at home. Programs focusing on supporting family caregivers for long-term conditions and education for family caregivers surrounding care for heart, circulatory, and blood conditions are suggested.

